# Expression profiles, biological functions and clinical significance of circRNAs in bladder cancer

**DOI:** 10.1186/s12943-020-01300-8

**Published:** 2021-01-04

**Authors:** Xiaoqi Yang, Tao Ye, Haoran Liu, Peng Lv, Chen Duan, Xiaoliang Wu, Kehua Jiang, Hongyan Lu, Ding Xia, Ejun Peng, Zhiqiang Chen, Kun Tang, Zhangqun Ye

**Affiliations:** 1 Department of Urology, Tongji Hospital, Tongji Medical College, Huazhong University of Science and Technology, Wuhan, China; 2 Department of Urology, Guizhou Provincial People’s Hospital, Guiyang, China; 3 Department of Urology, The Third Affiliated Hospital of Chongqing Medical University, Chongqing, China

**Keywords:** Circular RNA, Bladder cancer, ceRNA, Biomarker, Targeted therapy

## Abstract

Circular RNAs (circRNAs), which are single-stranded closed-loop RNA molecules lacking terminal 5′ caps and 3′ poly(A) tails, are attracting increasing scientific attention for their crucial regulatory roles in the occurrence and development of various diseases. With the rapid development of high-throughput sequencing technologies, increasing numbers of differentially expressed circRNAs have been identified in bladder cancer (BCa) via exploration of the expression profiles of BCa and normal tissues and cell lines. CircRNAs are critically involved in BCa biological behaviours, including cell proliferation, tumour growth suppression, cell cycle arrest, apoptosis, invasion, migration, metastasis, angiogenesis, and cisplatin chemoresistance. Most of the studied circRNAs in BCa regulate cancer biological behaviours via miRNA sponging regulatory mechanisms. CircRNAs have been reported to be significantly associated with many clinicopathologic characteristics of BCa, including tumour size, grade, differentiation, and stage; lymph node metastasis; tumour numbers; distant metastasis; invasion; and recurrence. Moreover, circRNA expression levels can be used to predict BCa patients’ survival parameters, such as overall survival (OS), disease-free survival (DFS), and progression-free survival (PFS). The abundance, conservation, stability, specificity and detectability of circRNAs render them potential diagnostic and prognostic biomarkers for BCa. Additionally, circRNAs play crucial regulatory roles upstream of various signalling pathways related to BCa carcinogenesis and progression, reflecting their potential as therapeutic targets for BCa. Herein, we briefly summarize the expression profiles, biological functions and mechanisms of circRNAs and the potential clinical applications of these molecules for BCa diagnosis, prognosis, and targeted therapy.

**Supplementary Information:**

The online version contains supplementary material available at 10.1186/s12943-020-01300-8.

## Background

Circular RNAs (circRNAs) are single-stranded closed-loop RNA molecules without terminal 5′ caps and 3′ poly(A) tails [1]. Although circRNAs were first discovered in viruses in 1976, they were initially regarded as functionless by-products of aberrant RNA splicing and consequently did not receive considerable scientific attention for decades [2, 3]. CircRNAs are produced from precursor mRNAs mainly by lariat-driven circularization and intron pairing-driven circularization, resulting in three types of circRNAs: exonic circRNAs (ecRNAs), exon-intron circRNAs (elciRNAs), and intronic circRNAs (ciRNAs) [4–7]. With the rapid development of high-throughput sequencing technologies, increasing numbers of differentially expressed circRNAs have been identified in normal and malignant human cells [8]. Numerous circRNAs exist widely in tissues, serum, and urine, and their expression profiles are cell type-specific, tissue-specific, or developmental stage-specific [9–12]. Increasing evidence suggests that circRNAs are involved in the occurrence and development of various diseases, such as cardiovascular diseases [13], diabetes [14], neurological dysfunction [15], and cancer [16–19]. In particular, circRNAs have been reported to play pivotal roles in the development and progression of cancer and might function as cancer biomarkers and novel therapeutic targets [20, 21]. CircRNAs perform regulatory roles at the transcriptional and posttranscriptional levels; for example, they modulate gene transcription [6], act as microRNA (miRNA) sponges [22], interact with RNA-binding proteins (RBPs) [23], and can be translated into peptides [24].

Bladder cancer (BCa) is the most common malignant tumour of the urinary system, and its incidence is increasing worldwide [25]. BCa is divided into three main pathological types: bladder urothelial carcinoma (BUC), squamous cell carcinoma and adenocarcinoma, with BUC accounting for > 90% of all cases of BCa [26]. To assign risk, BUC can be further categorized into muscle-invasive BCa (MIBC) and non-muscle-invasive BCa (NMIBC), with NMIBC accounting for approximately 75% of all cases [27]. Recently, the treatment of BCa has achieved great advances worldwide. Apart from traditional surgical resection, chemotherapy, and radiotherapy, immunotherapy is a promising method for BCa treatment [28, 29]. However, postoperative recurrence and distant metastasis make five-year survival rates for advanced BCa still low [30, 31]. Therefore, identifying potential therapeutic targets and biomarkers for BCa is of great importance.

An increasing number of studies have shown that differential expression of circRNAs is associated with the carcinogenesis and progression of BCa. In this review, we summarize the expression profiles, biological functions and mechanisms, and clinical significance of BCa-related circRNAs.

### Biogenesis, function, and study approaches of circRNAs

#### Biogenesis of circRNAs

CircRNAs are produced from pre-mRNAs and are thought to be the result of exon-skipping events. Although the specific mechanism of circRNA biogenesis is still unidentified, two widely accepted models of circRNA circularization can explain the back-splicing processes known as lariat-driven circularization and intron pairing-driven circularization [4]. In the lariat-driven circularization model, circularization requires covalent binding between the splicing donor and splicing acceptor to form an exon-containing lariat, resulting in the formation of ecRNAs [32]. In the intron pairing-driven circularization model, circularization is generated by base pairing between reverse complementary sequences. Alu repeats, originally characterized by the action of the Arthrobacterluteus restriction endonuclease, are repetitive complementary sequences located in flanking introns and are highly abundant and exist in more than 10% of the human genome [4, 33]. Introns consisting of Alu repeats are more likely to pair with each other, leading to circularization of exons and production of diverse circRNAs [34]. Unlike ecRNAs, elciRNAs retain introns that are not spliced out completely [6]. CiRNAs are generated from intron lariats that escape the process of intron debranching and degradation [5]. In addition, circRNA biogenesis has been reported to be regulated by a number of proteins, such as RBPs [23], enzymes [35], and transcription factors [36]. RBPs are crucial regulatory factors that interact with specific binding sites in flanking intronic sequences of precursor mRNAs to promote or suppress circRNA formation. For example, quaking (QKI) is an RBP that induces exon circularization and then facilitates the biogenesis of circRNAs when it binds to intronic QKI binding motifs [23]. In addition, another RBP, muscleblind (MBL), has been reported to interact with its own pre-mRNA, leading to the formation of circMBL [37]. Adenosine deaminase acting on RNA (ADAR1), a kind of RNA-editing enzyme, was reported to negatively regulate the formation of circRNAs by reducing the RNA pairing structure of flanking introns and backsplicing [35]. Moreover, the nuclear RNA helicase DHX9 can reduce the formation of circRNAs by downregulating Alu element-induced intron pairing [38]. Finally, the transcription factor Twist1 was found to bind the Cul2 promoter to selectively promote the expression of Cullin2 (Cul2) circular RNA during the epithelial–mesenchymal transition in hepatocellular carcinoma [36]. In brief, the biogenesis of circRNAs and the regulatory mechanisms involved in circularization remain vague. More research is needed to help us understand the circRNA circularization processes in depth.

#### Functions of circRNAs

CircRNAs were initially regarded as functionless by-products of aberrant RNA splicing [2, 3]. With the rapid development of high-throughput sequencing technologies, an increasing number of circRNAs have been found to be involved in physiological and pathological processes by acting as miRNA sponges [22], interacting with RBPs [23], regulating transcription or splicing [37, 39], and translating proteins [24]. Among these biological processes, circRNAs most commonly exert their function by sponging miRNAs in tumour cells. For example, ciRS-7 (circ_Cdr1as) serves as an miRNA sponge of miR-7, resulting in decreased miR-7 function and upregulation of miR-7 target genes [40]. In addition to acting as miRNA sponges, some circRNAs may also serve as protein sponges or decoys to interact with RBPs. For instance, circ_Foxo3 was found to block cell cycle progression by binding to the cell cycle proteins cyclin-dependent kinase 2 (CDK2) and cyclin-dependent kinase inhibitor 1 (p21) [41]. In addition, circ-PABPN1 was found to bind to human antigen R/ELAV-like protein 1 (HuR) and prevent HuR from binding to PABPN1 mRNA, resulting in the inhibition of PABPN1 translation [42]. Some circRNAs have also been identified to regulate gene transcription or selective splicing. Circ_EIF3J and circ_PAIP2 have been reported to promote the transcription of PAIP2 and EIF3J by interacting with U1 snRNPs [6]. Additionally, circ_Mbl was reported to compete with linear MBL mRNA for selective splicing [37]. Finally, increasing evidence has demonstrated that some circRNAs can exert their functions by translating proteins. Due to the absence of 5′-cap and 3′-poly(A) structures, circRNAs were initially considered to be untranslatable [43]. Recently, translatable circRNAs containing internal ribosome entry sites (IRESs) were found to be translated into proteins in a cap-independent manner [44–46]. For example, circ-ZNF609 was reported to be translated into a protein that controls myoblast proliferation [24]. In addition, circFXBW7 can be translated into a novel 21-kDa protein to suppress the tumorigenesis of glioma [47].

#### Approaches for circRNA studies

To date, genome-wide annotation of circRNAs, experimental validation of circRNAs, and overexpression/suppression of circRNAs are the main approaches to explore the functional implications of circRNAs. First, ribo-RNA-seq profiles rRNA-depleted total RNAs, including both poly(A) (linear) and nonpoly(A) (circular) RNAs. In addition, p(A)- RNA-seq profiles only non-poly(A) RNA. Ribo-RNA-seq or p(A)-RNA-seq combined with RNase R, which digests linear RNAs and preserves circRNAs, is more suitable for biochemical enrichment detection of circRNAs [48]. In addition, bioinformatic mapping was used to identify RNA-seq reads uniquely mapped to back-splice junctions (BSJs) via a number of algorithms [49]. In addition to RNA-seq profiling, microarray technology is also used for circRNA annotation [50]. Second, a series of experimental approaches, including PCR [51], northern blotting [5], and RNA fluorescence in situ hybridization (FISH) [6], are widely used to validate the existence of circRNAs [49]. Finally, overexpression/suppression of circRNAs are gain/loss of function used to annotate circRNAs’ function. Overexpression of circRNAs can be achieved in trans by overexpression plasmids, which contain circRNA-producing exons and their flanking intronic sequences with intronic complementary sequences [52]. In theory, manipulation of the endogenous promoter with the CRISPR/Cas9 genome engineering system or replacement of the weak intronic RNA pair with a strong one can lead to overexpression of both circular and linear RNAs from a gene locus in cis [49]. RNAi-mediated degradation [53] and the RNA-guided, RNA-targeting Cas13 system [54] represent strategies for circRNA knockdown. The CRISPR/Cas9 genome engineering system targeting circRNA-forming exons or disrupting intronic RNA pairs are strategies for circRNA knockout [55, 56]. In conclusion, improvements in methods to study circRNAs without affecting their residing genes and the wide employment of improved experimental approaches will be able to provide new insights into the biogenesis and functional implications of circRNAs in the future.

### Research on and discovery of circRNAs in BCa

**Fig. 1:**
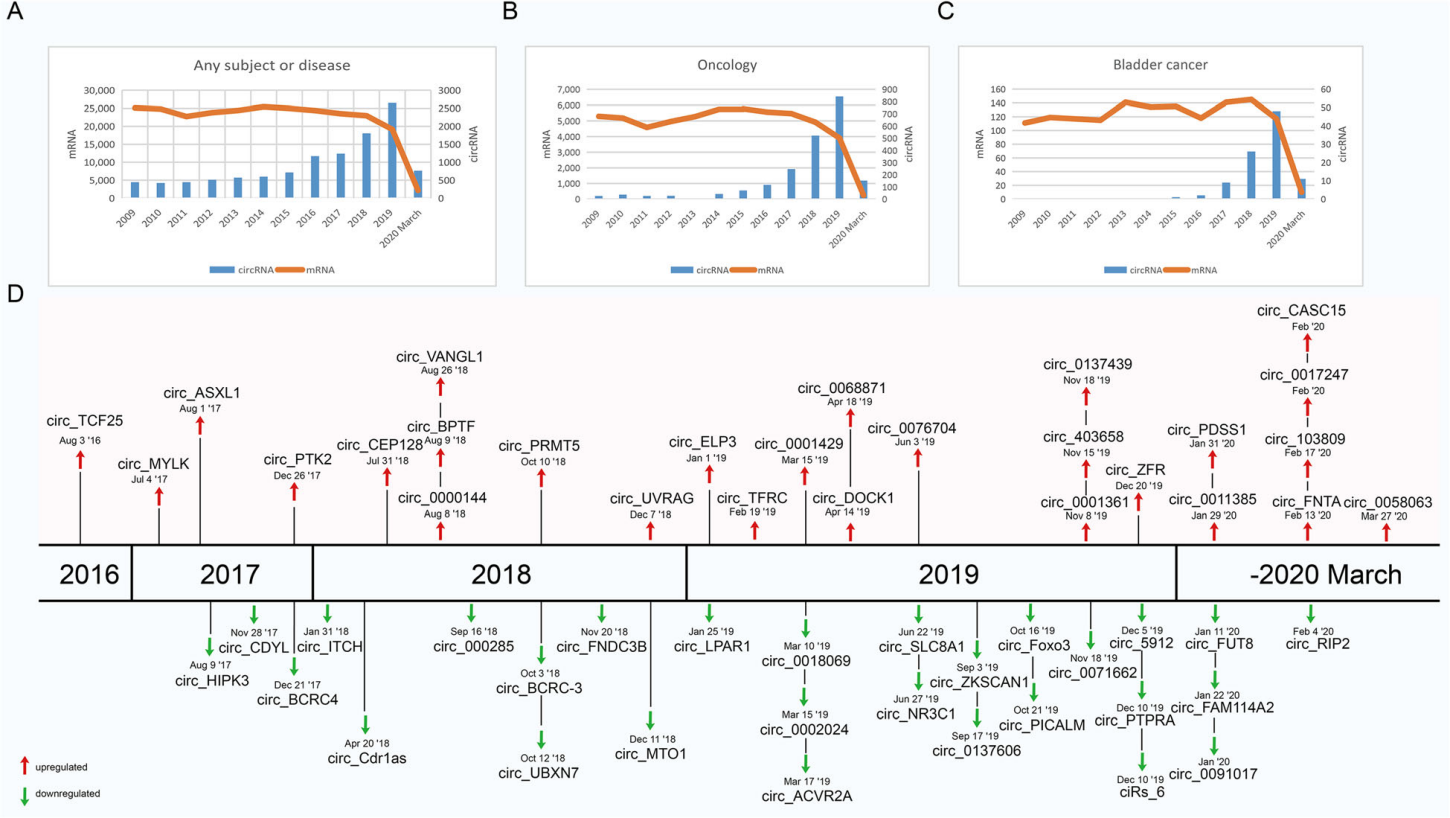
Research on and discovery of circRNAs in BCa. The amount of research, as quantified by the annual number of peer-reviewed publications, has been relatively stable for mRNAs (orange line) but not for circRNAs (blue bars) in the following categories: **a** overall, for any subject or disease; **b** oncology; and **c** BCa. **d** Increasing numbers of novel circRNAs were identified from 2016 to March 2020

A full review was performed using Web of Science to search for reports with the key words (“circular RNA” or “circRNA”) and (“bladder cancer” or “bladder urothelial carcinoma” or “bladder neoplasm” or “bladder tumor” or “bladder tumour”) published over the past 10 years (January 2009–March 2020). Research regarding the discovery and characterization of circRNAs has increased annually, while protein-coding gene (mRNA) discovery research has remained stable (Fig. 1a). Similar trends are observed in the contexts of oncology in general (Fig. 1b) and BCa specifically (Fig. 1c). These findings suggest a growing focus on circRNAs and their roles in tumorigenesis. Collectively, related research has resulted in the validation of 55 BCa-related circRNAs (27 upregulated and 28 downregulated) in the past 10 years (Fig. 1d).

A variety of methods have been developed to study the structures and functions of circRNAs. RNA sequencing (RNA-seq) [32] and microarray technology [50] are widely used for the identification of new circRNAs and the quantification of circRNA expression. Reverse transcription-polymerase chain reaction (RT-PCR) [51] and Northern blot [57] are two approaches used to further verify circRNAs. In addition, FISH can be used to analyse the subcellular localization of circRNAs [58].

To better study the biological functions and applications of circRNAs, numerous circRNA-associated public databases have recently been developed to facilitate circRNA analyses. These online databases are currently used for circRNA identification, prediction, localization, characterization and functional analysis and provide tools for investigating the interactions of circRNAs with targets. For example, CircBase contains circRNA information from different species [59]. CircRNADisease and Circ2Traits provide disease-associated circRNA annotations [60, 61]. Other databases and their common uses are listed in Table S1.

### CircRNA expression profiles in BCa

With the rapid development of high-throughput sequencing technologies, a large number of novel dysregulated circRNAs have been identified in BCa cell lines and tissues, most of which are differentially expressed between BCa tissues and adjacent normal tissues, indicating the important roles of these circRNAs in BCa development and progression. Primary expression profiles obtained via ribosomal RNA-depleted RNA-seq and circRNA microarrays have been widely employed for the discovery of novel circRNAs [32, 59, 62]. For example, according to circRNA microarray data from 4 paired BCa tissues and adjacent normal bladder tissues, Zhong et al. identified 3243 circRNAs in total and 469 circRNAs that were differentially expressed in BCa compared with normal tissues, 285 of which were significantly upregulated, while 184 were downregulated [63]. Li et al. identified 16,353 circRNAs that were expressed in 3 paired BCa and adjacent normal tissues, 571 of which were differentially expressed; 47 circRNAs were significantly upregulated, and 524 circRNAs were downregulated [64]. Zheng et al. identified 67,358 circRNAs that were expressed in 1 pair of BCa and adjacent normal bladder tissues, and circ_HIPK3 was verified to directly bind to miR-124 and inhibit miR-124 activity [65]. In another study, RNA-seq data from BCa tissues of 9 different grades and adjacent normal bladder tissues revealed a total of 316 (205 upregulated and 111 downregulated) and 244 (109 upregulated and 135 downregulated) dysregulated circRNAs in high-grade BCa vs. normal tissue and in high-grade BCa vs. low-grade BCa, respectively; 42 of these circRNAs overlapped [66]. In addition, secondary bioinformatic analyses based on Gene Expression Omnibus (GEO) databases are frequently performed to identify differentially expressed circRNAs [67]. By performing a comprehensive bioinformatics analysis of RNA-seq data from 457 NMIBC samples, Okholm et al. identified 15,223 unique circRNAs that were supported by at least two reads in at least two different samples, and 113 abundant circRNAs were differentially expressed between high- and low-risk tumour subtypes; furthermore, the expression of 13 circRNAs correlated with progression [68]. Among 11 studies, the microarray dataset GSE92675 from the platform GPL19978 was the most commonly used database for secondary bioinformatic analyses intended to identify novel circRNAs for further research [69–79]. BCa-related circRNAs identified by RNA-seq and microarray analyses are listed in Table 1.

**Table 1:** Overview of circRNAs identifed by RNA sequencing and microarrays in BCa

Sample	Special treatment	Detection Method	GEO database	Data source	total circRNA	Number of circRNA differently expressed (fold change ≥2)	circRNAs validated by qRT-PCR	Ref./PMID
4 paird BCa and BCN tissues	RNAse R	CircRNA microarray	GSE92675	CircRNA microarray	3243	469 (285 upregulated, 184 downregulated)	6	27484176
3 paird BCa and BCN tissues	rRNA-depleted and RNase R	RNA-seq	GSE97239	RNA-seq	16,353 (6154)	571 (47 upregulated, 524 downregulated)	circ_HIPK3	28794202
1 paird BCa and BCN tissues	rRNA-depleted	RNA-seq	GSE77661	RNA-seq	67,358(27296)	/	circ_HIPK3	27050392
4 paird BC and BCN tissues	/	RNA-seq	/	RNA-seq	/	59(7 upregulated, 52 downregulated)	hsa circ 0018069	30984788
2 paird BCa and BCN tissues	RNAse R	RNA-seq	/	RNA-seq	6834	567	40	30745833
5 paird BCa and BCN tissues	rRNA and linear RNA-depleted	RNA-seq	/	RNA-seq	88,732(62,788)	56 (14 upregulated, 42 downregulated)	2	29151929
4 paird BCa and BCN tissues	rRNA-depleted	RNA-seq	/	RNA-seq	/	118 (34 upregulated, 84 downregulated)	3	30025927
9 different grades of BCa and BCN tissues	/	RNA-seq	/	RNA-seq	/	244 (H vs L), 316 (H vs N), 42 circRNAs overlapped	7	31545480
BCa 5637, T24 and SV-HUC-1 cell lines	/	CircRNA microarray	/	CircRNA microarray	/	/	circ_CASC15	31072448
10 paird BCa and HC urine samples	Rnase R	CircRNA microarray	/	CircRNA microarray	/	86 (53 upregulated, 33 downregulated)	circ_0137439	31777254
3 pairs of BCSCs and BCNSCs samples	/	CircRNA microarray	/	CircRNA microarray	4451	127 (113 upregulated, 14 downregulated)	circ_103809	32065779
3 paird BCa and BCN tissues	/	CircRNA microarray	/	CircRNA microarray	/	734 (478 upregulated, 256 downregulated)	8	30983072
3 paird BCa and BCN tissues	Rnase R	CircRNA microarray	GSE112719	CircRNA microarray	/	80 (37 upregulated, 43 downregulated)	circ_101320	30305293
3 paird BCa and BCN tissues	/	CircRNA microarray	/	CircRNA microarray	1038	/	7	29558461
457 NMIBC samples	/	RNA-Seq	/	Bioinformatics analysis	15,223	/	13	29263845
4 paird BCa and BCN tissues	/	CircRNA microarray	GSE92675	Bioinformatics analysis	/	469 (285 upregulated, 184 downregulated)	circ_MYLK	28687357
3 paird BCa and BCN tissues	/	/	GSE97239, GSE92675	Bioinformatics analysis	/	18 (5 upregulated, 13 downregulated)	3	31169020
4 paird BCa and BCN tissues	/	CircRNA microarray	GSE92675	Bioinformatics analysis	/	200	hsa_circ_0000144	30098434
4 paird BCa and BCN tissues	/	CircRNA microarray	GSE92675	Bioinformatics analysis	/	408	circ_0058063	30362519
4 paird BCa and BCN tissues	/	CircRNA microarray	GSE92675	Bioinformatics analysis	/	89 (66 upregulated, 23 downregulated)	circ_0001429	30909190
4 paird BCa and BCN tissues	/	CircRNA microarray	GSE92675	Bioinformatics analysis	/	433 (264 upregulated, 169 downregulated)	circ_CEP128	30134837
4 paird BCa and BCN tissues	/	CircRNA microarray	GSE92675	Bioinformatics analysis	3423	433 (264 upregulated, 169 downregulated)	circ_CEP128	30939216
4 paird BCa and BCN tissues	/	CircRNA microarray	GSE92675	Bioinformatics analysis	/	312 (195 upregulated, 117 downregulated)	circ_0058063	32181485
4 paird BCa and BCN tissues	/	CircRNA microarray	GSE92675	Bioinformatics analysis	/	/	circ_VANGL1	30146736
4 paird BCa and BCN tissues	/	CircRNA microarray	GSE92675	Bioinformatics analysis	3243	469 (285 upregulated, 184 downregulated)	/	27363013
4 paird BCa and BCN tissues	/	CircRNA microarray	GSE92675	Bioinformatics analysis	/	428 (261 upregulated, 167 downregulated)	hsa_circ_0011385	32015691

*circRNAs* circular RNAs, *BCa* bladder cancer, *BCN* bladder cancer tissues paired adjacent normal bladder tissues, *H* high-grade bladder cancer, *L* low-grade bladder cancer, *N* normal tissue, *HC* healthy controls, *BCSCs* bladder cancer stem cells, *BCNSCs* bladder cancer non-stem cells, *NMIBC* non muscle-invasive bladder cancer

For microarray or RNA-seq data analyses, paired t tests were performed to analyse significant differences. The false discovery rate (FDR) was applied to determine the *P*-value threshold, and an FDR < 0.05 was recommended. CircRNAs (fold changes ≥ 2.0 and *P*-values < 0.05) have been reported to be significantly differentially expressed [63, 64]. For RT-PCR or Northern blotting, β-actin or GAPDH was used as a reference gene. Mean values, median expression levels, or concrete data are used as cut-off values [80–83]. No unified standards are available to determine thresholds for circRNA detection.

### Biological functions and molecular mechanisms of circRNAs in BCa

#### CircRNAs regulate the hallmarks of cancer

**Fig. 2:**
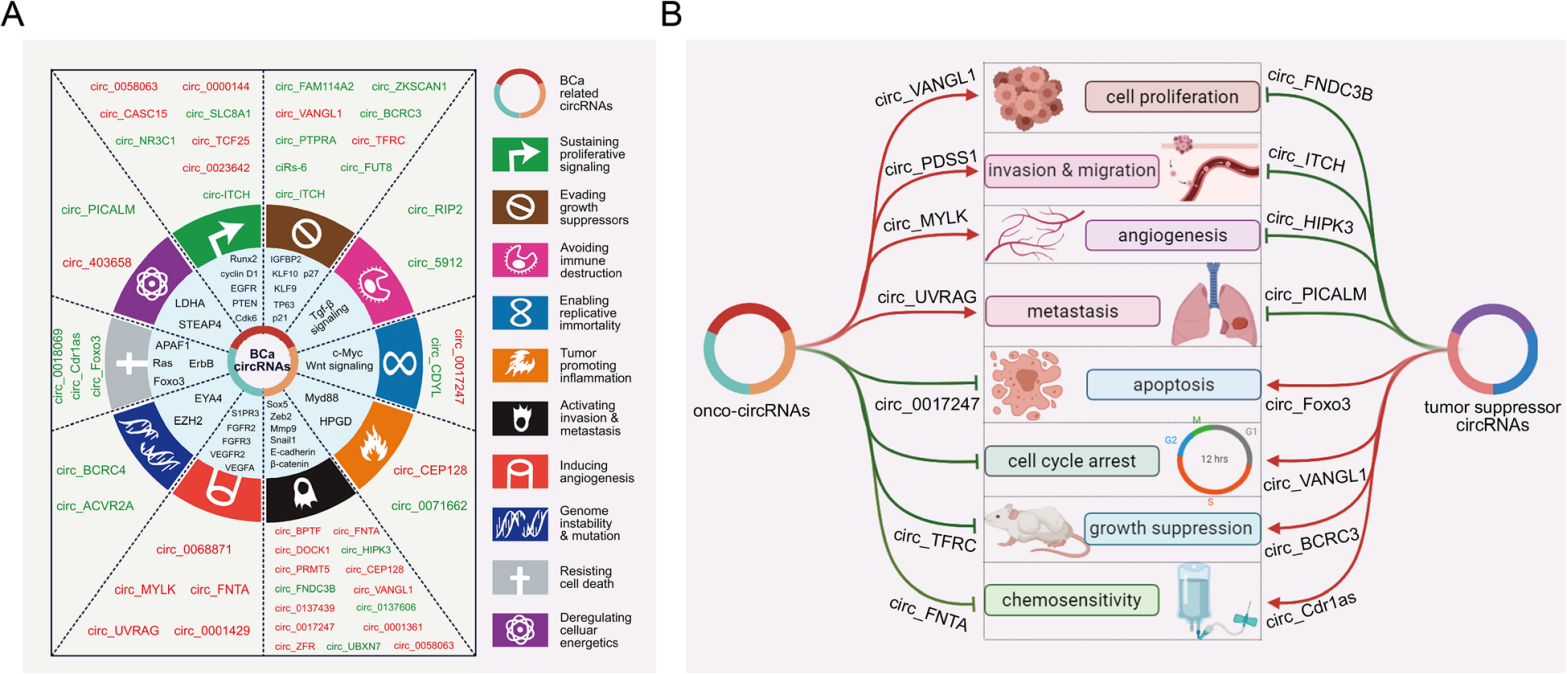
The relationship between circRNAs and BCa. **a** CircRNAs are associated with the hallmarks of BCa. **b** CircRNAs regulate cell proliferation, apoptosis, invasion, migration and metastasis, angiogenesis, and cisplatin chemoresistance in BCa cells

In 2011, Hanahan and Weinberg proposed ten hallmarks of cancer that result in the progressive conversion of normal cells into cancerous cells [84]. Here, we briefly summarize the well-known circRNAs involved in the essential stages of tumourigenesis and progression in BCa to examine the correlations between circRNAs and the hallmark features of cancer (Fig. 2a).

Recently, several oncogenic and antioncogenic circRNAs have been discovered to regulate cell proliferation, tumour growth suppression, cell cycle arrest, apoptosis, invasion, migration, metastasis, angiogenesis, and cisplatin chemoresistance in BCa cells (Fig. 2b and Table 2).

**Table 2:** Dysregulated circRNAs in BCa

Name	CircBase ID	Sponge target	Gene	Function	Types of BCa tissues and BCa cell lines	Ref./PMID
upregulated						
circ_0058063	hsa_circ_0058063	miR-486-3p	FOXP4	promoted cell proliferation, invasion, and inhibited apoptosis	94 cases of BCa and the non-cancerous normal tissues; 5637, BIU-87 and RT-112 cell lines	32181485
circ_0058063	hsa_circ_0058063	miR-145-5p	CDK6	promoted cell proliferation and migration, inhibited cell apoptosis, and decreased cell cycle arrest	25 pairs of BCa tissues and adjacent normal tissues; T24 and J82 cell lines	30362519
circ_0017247	hsa_circ_0017247	/	Wnt/β-catenin	promoted cell proliferation and tumor formation, inhibited apoptosis, and decreased cell cycle arrest	50 BCa tissues and adjacent tissues; UM-UC3, SW780, BIU and J82 cell lines	32096177
circ_103809	hsa_circ_0072088	miR-511	/	promoted the self-renewal, migration and invasion	3 pairs of BCa tissues and adjacent normal bladder tissues; T24 and EJ cell lines	32065779
circ_FNTA	hsa_circ_0084171	miR-370-3p	FNTA	promoted invasion and decreased chemosensitivity to cisplatin	41 BCa tissues and adjacent normal bladder tissues; T24, J82, 5637, and UMUC3 cell lines	32052578
circ_PDSS1	hsa_circ_0093398	miR-16	/	promoted cell proliferation, invasion and migration	72 BCa and adjacent healthy tissues; HT-1197 and UMUC3 cells	31868205
circ_403658	hsa_circ_0004383	/	LDHA	promoted cell growth and invasion	123 BCa tissues and the matched adjacent tissues; SW780, 5637, T24, J82 and RT4 cell lines	31814891
circ_0137439	hsa_circ_0137439	miR-142-5p	MTDH	promoted cell proliferation, migration, and metastasis	116 bladder cancer urine samples and 30 normal samples; T24 and 5637 cell lines	31777254
circ_VANGL1	hsa_circ_0002623	miR-605-3p	VANGL1	promoted cell proliferation, migration, and invasion	87 BCa tissues and 37 normal adjacent tissues; T24 and EJ cells	30146736
circ_VANGL1	hsa_circ_0002623	miR-1184	IGFBP2	promoted cell proliferation, migration, and invasion	60 BCa and corresponding paracancerous tissue; J82, T24, EJ, RT-4, UM-UC-3, and TCC cell lines	31758655
circ_ZFR	hsa_circ_0072088	miR-377	ZEB2	promoted cell growth, migration and invasion, and decreased cell cycle arrest and apoptosis	104 pairs of BCa tissues and adjacent normal tissues; UMUC3, T24, J82, 5637, SW780, EJ and BIU87 cell lines	31746333
circ_0001361	hsa_circ_0001361	miR-491-5p	MMP9	promoted cell invasion and metastasis	69 pairs of BCa tissues and matched adjacent normal bladder epithelial tissues; SV-HUC-1, EJ, UMUC3, RT4, and 5637 cell lines	31705065
circ_UVRAG	hsa_circ_0023642	miR-223	FGFR2	promoted proliferation, migration, tumor formation, and metastasis	T24, EJ, J82, UM-UC-3, TCC, and RT-4 cell lines	30387298
circ_0023642	hsa_circ_0023642	miR-490-5p	EGFR	promoted cell invasion and metastasis	J82 and UMUC3 cell lines	31455760
circ_CASC15	hsa_circ_0075828	miR-1224-5p	CREB1	promoteed cell proliferation	67 pair BCa tissues and matched para-carcinoma tissues; 5637, and T24 cell lines	31072448
circ_0068871	hsa_circ_0068871	miR-181a-5p	FGFR3	promoted cell proliferation and migration	32 BCa and adjacent normal tissue; T24, UMUC3, EJ, and J82 cell lines	30999937
circ_DOCK1	hsa_circ_0020394	miR-132-3p	Sox5	promoted cell proliferation, migration, and tumour growth	23 BC tissue specimens and 32 normal bladder tissues; BIU-87, EJ-m3, T24 and 5673 cell lines	30983072
circ_CEP128	hsa_circ_0102722	miR-145-5p	SOX11	promoted cell proliferation, and decreased cell apoptosis and cell cycle arrest	10 pairs of BCa tissues and adjacent bladder tissues; RT-112, 5637, BIU-87, TCCSUP and HEK293T cell lines	30134837
circ_CEP128	hsa_circ_0102722	miR-145-5p	Myd88	promoted cell proliferation and migration, and decreased cell apoptosis and cell cycle arrest	40 BCa specimens and blood samples; 293T, J82 and T24 cell lines	30939216
circ_0001429	hsa_circ_0001429	miR-205-5p	VEGFA	enhanced cell propagation and metastasis, reduced cell apoptosis, and promoted tumor growth and lung metastasis	20 pairs of BCa tissues and paired adjacent normal bladder tissues; T24 cells and 5637 cells	30909190
circ_TFRC	has_circ_0001445	miR-107	TFRC	promoted the invasion, proliferation and tumor growth, contributed to an EMT phenotype	57 BCa patients tissues compared with adjacent normal patients tissues, EJ, T24, 5637, UMUC3, BIU87, J82, and SW780 cell lines	30782157
circ_ELP3	hsa_circ_0001785	/	/	promoted cell proliferation, and reduced apoptosis and chemosensitivity to cisplatin	18 pairs of tissue samples and 30 bladder cancer samples; T24 and 5647 cells	30745833
circ_PRMT5	hsa_circ_0031250	miR-30c	SNAIL1/E-cadherin	promoted cell EMT	119 UCB tissues with matched adjacent normal bladder tissues; T24, TCC-SUP, 5637, and UM-UC-3 cell lines	30305293
circ_BPTF	hsa_circ_0000799	miR-31-5p	RAB27A	promoted cell migration and invasive, and tumor growth	72 pairs of specimens of BCa tissues and adjacent noncancerous tissues; UM-UC-3 and T24 cell lines	30103209
circ_0000144	hsa_circ_0000144	miR-217	RUNX2	promoted cell proliferation and invasion	21 pairs of BCa tissues and adjacent normal tissues; T24, EJ, UMUC3, RT4 and 253J cell lines	30098434
circ_PTK2	hsa_circ_0003221	/	/	promoted the proliferation and migration	40 pairs of BCa tissue and blood samples. T24 and 5637 cell lines	29125888
circ_MYLK	hsa_circ_0002768	miR-29a	VEGFA/VEGFR2	accelerated cell proliferation, migration, tube formation, and promoted EMT	32 pairs bladder carcinomas and matched para-carcinomas; EJ, T24, 5673 and BIU-87 cell lines	28687357
circ_TCF25	hsa_circ_0041103	miR-103a-3p/miR-107	CDK6	promoted proliferation and migration	40 pairs bladder carcinoma tissue and matched para-carcinoma tissues; T24 and EJ cell lines	27484176
downregulated						
circ_FUT8	hsa_circ_0003028	miR-570-3p	KLF10	inhibited migration, invasion, and metastasis	145 BCa tissues and 50 matched adjacent normal bladder tissues; T24, SV-HUC-1, and UM-UC-3 cell lines	32072011
circ_RIP2	hsa_circ_0005777	miR-1305	Tgf-β2/smad3	promoted migration, invasion, clone formation and EMT	45 paired BCa and the adjacent normal tissues, 58 bladder cancer tissues, 5637 and UM-UC-3 cell lines	32019579
circ_FAM114A2	hsa_circ_0001546	miR-762	ΔNP63/TP63	inhibited migration, invasion and proliferation	31 BCa tissues and paired adjacent noncancer tissues; T24, J82, 5637, and 293T cell lines	31969560
circ_0091017	hsa_circ_0091017	miR-589-5p	/	inhibited cell proliferation, migration and invasiveness	40 pairs of BCa tissues and normal adjacent tissues; 5637, EJ, T24, UMUC-3, and RT4 cell lines	31957821
ciRs_6	hsa_circ_0006260	miR-653	March1	suppressed cell growth and increased cell cycle arrest	45 paired bladder cancer and the adjacent normal tissues, 58 bladder cancer tissues; 5637 and UM-UC-3 cell lines	31819015
circ_5912	hsa_circ_0005912	/	TGF-β signaling	suppressesed cell proliferation, invasion and migration	58 BCa tissues and the matched adjacent tissues; T24 and SW780 cell lines	31808751
circ_PTPRA	hsa_circ_0006117	miR-636	KLF9	inhibited cell proliferation and tumor growth	104 BCa specimens, 64 matched BC and adjacent normal specimens; T24 and UM-UC-3 cell lines	31821171
circ_Foxo3	hsa_circ_0006404	miR-191-5p	/	promoted cell apoptosis	30 BCa tissues and adjacent normal bladder tissues; T24, UM-UC-3 and J82 cell lines	31802888
circ_0071662	hsa_circ_0071662	miR-146b-3p	HPGD/NF2	suppressed cell proliferation and invasion	97 BCa tissues and matched adjacent normal tissues; BIU-87, T-24, EJ-28 and J82 cell lines	31757227
circ_PICALM	hsa_circ_0023919	miR-1265	STEAP4	inhibited cell invasion and metastasis	168 BCa samples and 40 corresponding adjacent normal tissue samples; T24, UM-UC-3, J82, RT-4, and HEK-293T cell lines	31648990
circ_0137606	hsa_circ_0137606	miR-1231	PHLPP2	suppressed cell proliferation and metastasis	13 high-grade BCa, low-grade BCa and a normal controlpatients tissues; T24 and SV-HUC-1 cell lines	31545480
circ-ZKSCAN1	hsa_circ_0001727	miR-1178-3p	p21	inhibited cell proliferation, migration, invasion and metastasis	68 BCa tissues and the matched normal tissues; T24, UM-UC-3, 5637, and EJ cell lines	31481066
circ_NR3C1	hsa_circ_0001543	miR-27a-3p	cyclin D1	inhibited cell proliferation, cell cycle progression, and tumor growth	42 pairs of BCa tissues and adjacent normal bladder tissues; T24, EJ, UMUC3, J82, and 5637 cell lines	31255724
circ_SLC8A1	hsa_circ_0000994	miR-130b/miR-494	PTEN	inhibited cell migration, invasion and proliferation	70 pairs of human bladder cancer tissues compared with their adjacent normal tissues; 5637, T24, J82, EJ, UMUC, and RT4 cell lines	31228937
circ_Cdr1as	hsa_circ_0001946	miR-1270	APAF1	induced cell apoptosis and enhanced chemosensitivity to cisplatin	160 BCa tissues; TCCSUP, 5367, T24 and EJ cell lines	31131537
circ_Cdr1as	hsa_circ_0001946	miR-135a	/	inhibited cell proliferation, invasion and migration, and tumour growth	94 pairs of BCa tissues and adjacent normal tissues; EJ and T24 cell lines	29694981
circ_ACVR2A	hsa_circ_0001073	miR-626	EYA4	suppressed cell proliferation and metastasis	50BC tissues and matched adjacent normal epithelial tissues; T24, UM-UC-3, RT4, J82, 5637, HT-1376, and TCCSUP cell lines	31101108
circ_0002024	hsa_circ_0002024	miR-197-3p	/	suppressed cell proliferation, migration, and invasion	20 BCa and normal samples; EJ, 5637, T24, and UMUC-2 cell lines	30972190
circ_CDYL	hsa_circ_0008285	/	C-MYC	inhibited cell growth and migration	30 pairs of BCa tissues and paired surrounding normal bladder tissues; EJ and T24T cells	30968727
circ_LPAR1	hsa_circ_0087960	miR-762	/	inhibited cell invasion and metastasis	125 BCa tissues and 68 paired cancer tissues and adjacent non-tumorous tissues; 5637 and T24 cell lines	30867795
circ_MTO1	hsa_circ_0007874	miR-221	/	inhibited cell EMT and metastasis	117 bladder cancer tissues and the matched adjacent tissues; UMUC3, SVHUC1, T24, J82 and 5637 cell lines	30551873
circ_FNDC3B	hsa_circ_0006156	miR-1178-3p	G3BP2	inhibited cell proliferation, migration, invasion, tumorigenesis and metastasis	82 BCa tissues and 56 pairs of BCa tissues and adjacent noncancerous tissues; T24 and UM-UC-3 cell lines	30458784
circ_UBXN7	hsa_circ_0001380	miR-1247-3p	B4GALT3	inhibited cell proliferation, migration, invasion, and tumor growth	84 cases of BCa tissues including 30 paired BC tissues and adjacent nontumor tissues; SV-HUC-1, T24 and UM-UC-3 cell lines	30312173
circ_HIPK3	hsa_circ_0000284	miR-558	HPSE	inhibited cell migration, invasion, and angiogenesis, tumor growth, and metastasis	44 pairs of bladder cancer tissues and paired adjacent normal bladder tissues; UMUC3, and T24 cell lines	28794202
circ_BCRC3	hsa_circ_0001110	miR-182-5p	p27	inhibited cell proliferation, and impairs tumor growth, and increased cell cycle arrest	47 BCa tissues and their adjacent normal bladder tissues; EJ,T24, and SV-HUC-1 cell lines	30285878
circ_BCRC4	hsa_circ_0001577	miR-101	EZH2	promoted cell apoptosis and inhibited cell viability	24 pairs of fresh bladder cancer tissues and surrounding normal adjacent bladder tissues; UMUC3 and T24 cell lines	29270748
circ_ITCH	hsa_circ_0001141	miR-17, miR-224	p21, PTEN	inhibited cell proliferation, migration, invasion and metastasis, induced cell cycle arrest and cell apoptosis	72 pairs of BCa tumor and normal tissues; UMUC3, T24, J82, 353J, 5637, TCC, EJ and RT4 cell lines	29386015

*circRNAs* circular RNAs, *BCa* bladder cancer, *EMT* epithelial to mesenchymal transition

#### Cell proliferation

**Fig. 3:**
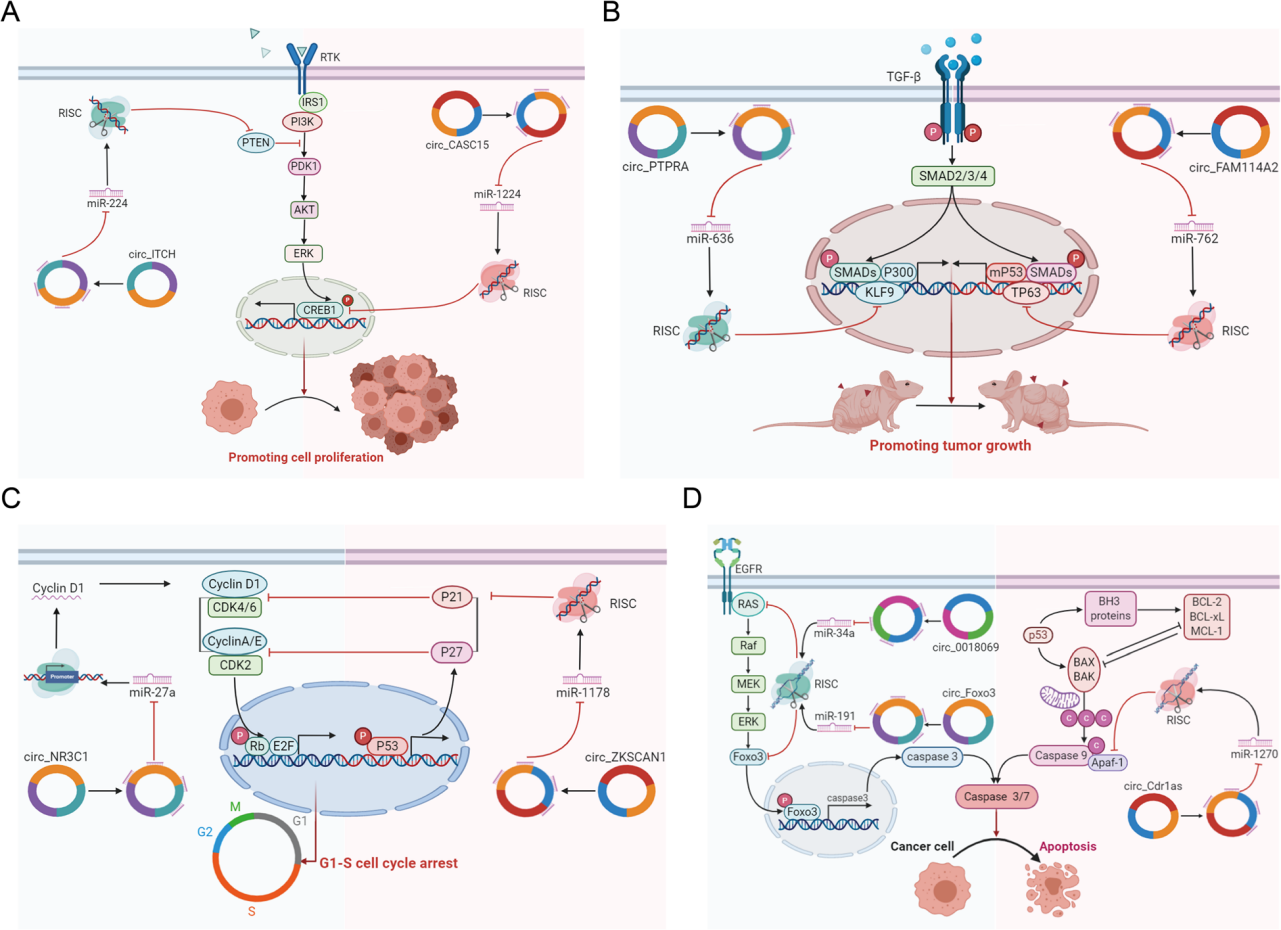
CircRNAs regulate cell proliferation, tumour growth suppression, cell cycle arrest, and apoptosis in BCa cells. **a** Roles of circRNAs in cell proliferation. **b** Roles of circRNAs in tumour growth suppression. **c** Roles of circRNAs in cell cycle arrest. **d** Roles of circRNAs in apoptosis

Tumour cells can sustain active proliferative states via activation of cell proliferation signalling pathways [84]. The PI3K/Akt/CREB signalling pathway is an important regulatory pathway of cell proliferation [85]. Circ_CASC15, derived from the CASC15 gene, promotes cell proliferation by acting as a miR-1224 sponge to activate oncogenic CREB1 expression in BCa [86]. Phosphatase and tensin homologue (PTEN), a negative regulator of the PI3K/Akt pathway, is highly involved in BCa progression [87]. Lu et al. found that circ_SLC8A1 inhibits BCa cell proliferation, migration, and invasion by upregulating PTEN expression [88]. Circ_ITCH, generated from several exons of itchy E3 ubiquitin protein ligase, suppresses cell proliferation by sponging miR-224 to increase the expression of PTEN in BCa [89] (Fig. 3a). Consistent with the results regarding BCa, circ_ITCH has also been reported to be downregulated and to suppress cell proliferation by inhibiting the Wnt/beta-catenin pathway in lung cancer [90], colorectal cancer [91], and oesophageal squamous cell carcinoma [92]. Hsa_circ_0000144, which is produced through back-splicing of the SLAMF6 first intron, facilitates BCa cell proliferation by upregulating the expression of RUNX2, which promotes cellular malignancy in BCa [71, 93]. Liang et al. also suggested that circ_0058063 facilitates BCa cell proliferation and invasion via the circ_0058063/miR-486-3p/FOXP4 axis [76]. Circ_0071662 has been identified to suppress BCa cell proliferation and invasion by upregulating the tumour suppressor genes HPGD and NF2 [94]. Song et al. revealed that the hsa_circ_0137439/miR-142-5p/MTDH axis contributes to the promotion of BCa cell proliferation and migration [81]. In addition, hsa_circ_0091017 has been found to inhibit BCa cell proliferation and migration [95]. Yu et al. proposed that circ_PDSS1 may promote proliferation, invasion and migration by inhibiting the tumour suppressor miR-16 [96]. Circ_PTK2 has also been reported to promote BCa cell proliferation and migration [97].

Apart from sustaining proliferative signals, cancer cells continuously evade the growth-suppressive effects of the tumour suppressor pathway [84]. TP63 is a member of the p53 family of transcription factors, and its ΔNp63 isoform is the major tumour-suppressing isoform in BUC cell lines and primary tumours [98]. Liu et al. reported that circ_FAM114A2 inhibits tumour growth by regulating ΔNP63 [99]. In addition, the tumour suppressor Krüppel-like factor 9 (KLF9) has been reported to inhibit tumour growth by modulating p53 [100]. In one investigation, He et al. found that circ_PTPRA inhibits BCa cell proliferation in vitro and tumour growth in vivo by upregulating KLF9 [101] (Fig. 3b). Circ_BCRC3 has also been identified to function as a tumour suppressor, inhibiting BCa tumour growth through the miR-182-5p/p27 axis [102]. Su et al. showed that ciRs-6 suppresses BCa growth by elevating the expression of March1, a tumour suppressor gene that encodes an E3 ubiquitin-ligating enzyme [103]. Furthermore, ZEB2 has been reported to play oncogenic roles in BCa [104, 105]. According to Zhang et al., circ_ZFR facilitates BCa cell growth, migration and invasion by upregulating the expression of this gene [82].

Additionally, dysregulation of cell cycle regulators contributes to limitless tumour cell growth and proliferation [84]. C-MYC and C-MYC-induced genes play crucial roles in cell cycle control and cell growth [106]. For example, circ_CDYL induces cell cycle arrest by downregulating C-MYC and C-MYC-induced gene expression in BCa cells [107]. Cyclin D1, a key cell cycle-related protein, is believed to regulate the G1-to-S phase transition [108]. As reported by Zheng et al., circ_NR3C1 can induce G0/G1 arrest by suppressing cyclin D1 expression and subsequently inhibits cell cycle progression in BCa [109]. P21, a direct regulator of the cell cycle, plays a vital role in inducing growth arrest in the G1 phase by suppressing the activity of cyclin D-CDK2/4 complexes [110]. Bi et al. proposed that circ_ZKSCAN1 acts as a tumour suppressor to promote cell cycle arrest via the circ_ZKSCAN1/miR-1178-3p/p21 axis [111]. Circ_Cdr1as has also been found to mediate cell cycle arrest to exert anti-oncogenic functions in BCa cells by restoring p21 activity [112] (Fig. 3c). Among cell cycle-related CDKs, CDK6 has been identified as a major oncogenic driver of progression from G1 phase to S phase [113]. Circ_TCF25 promotes proliferation and migration by increasing CDK6 expression [63]. Sun et al. also discovered that circ_0058063 enhances BCa cell proliferation and migration abilities via the circ_0058063/miR-145-5p/CDK6 pathway in BCa [72].

#### Apoptosis

Apoptosis, autophagy, and necrosis are major mechanisms leading to controlled cell death that are strictly controlled by tumour cells [114]. Tumour cells can evade apoptosis, enabling them to achieve immortality. Apoptosis protease-activating factor-1 (APAF-1) is a key regulatory factor that interacts with cytochrome c released from the mitochondria, thus activating the caspase cascade to execute apoptosis [115]. Circ_Cdr1as, also known as ciRS-7 or CDR1NAT, has been identified to induce apoptosis of BCa cells by elevating APAF1 expression [116]. Forkhead box transcription factor class O3 (FOXO3) is another key factor that participates in apoptotic processes [117]. Wang et al. showed that circ_Foxo3 facilitates FOXO3-mediated apoptosis through miR-191-5p signalling [118]. Proapoptotic effects of circ_Foxo3 have also been observed in breast carcinoma biopsies and in cancer cell lines [119]. Consistent with these findings, Li et al. found by KEGG analysis that hsa_circ_0018069 may mediate the Foxo signalling pathway to exert anticancer effects [80] (Fig. 3d). In contrast, Wu et al. showed that circ_CEP128 promotes cell proliferation and suppresses apoptosis in the context of BCa by targeting SOX11 [74]. In another study, circ_CEP128 was illustrated to increase cell proliferation and inhibit apoptosis via the miR-145-5p/MYD88/MAPK signalling pathway [75]. According to Li et al., circ_BCRC4 enhances apoptosis through miR-101/EZH2 signalling [120].

#### Invasion, migration and metastasis

**Fig. 4:**
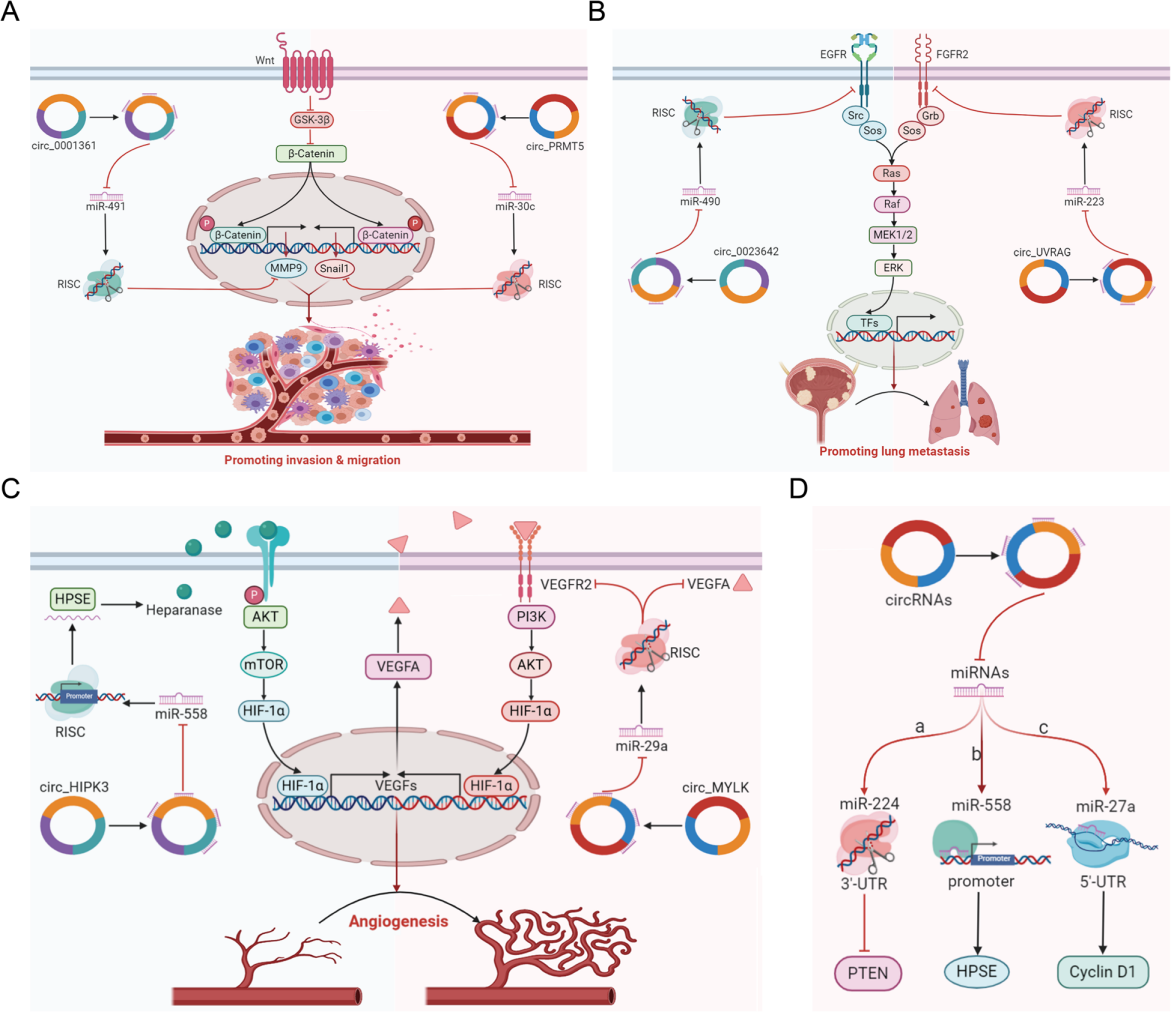
CircRNAs regulate invasion, migration and metastasis and angiogenesis in BCa cells and the molecular mechanisms of circRNAs in BCa. **a** Roles of circRNAs in invasion and migration. **b** Roles of circRNAs in lung metastasis. **c** Roles of circRNAs in angiogenesis. **d** Molecular mechanisms of circRNAs in BCa

Invasion, migration and metastasis of tumour cells into lymphatic and blood vessels for dissemination into the circulation eventually results in tumour colonization of distant organs [121]. MMP9, a member of the zinc-dependent endopeptidase family, plays crucial roles in invasion and migration by degrading the extracellular matrix in BCa [122, 123]. Liu et al. reported that circ_0001361, which is derived from two exons of the FNDC3B gene, increases MMP9 expression to promote BCa cell invasion and metastasis [124]. In addition, epithelial-mesenchymal transition (EMT) is an important mechanism for tumour invasion and metastasis [125]. Chen et al. revealed that circPRMT5 regulates the SNAIL1/E-cadherin-induced EMT pathway to promote BUC cell invasion and migration [62] (Fig. 4a). Su et al. indicated that circ_5912 suppresses the invasion and migration of BCa cells via the TGF-β2-induced EMT signalling pathway [126]. He et al. further revealed that circ_FUT8 suppresses the invasion and migration of BCa cells by regulating Slug and EMT [127]. Moreover, circ_RIP2 promotes BCa proliferation, invasion and migration by inducing EMT via activation of the miR-1305/TGF-β2/smad3 pathway [128]. Circ_TFRC has been reported to upregulate the proliferative and invasive abilities of BCa cells by activating the EMT signalling pathway [129]. In addition, FOXP4 promotes the migration and invasion of breast cancer cells via EMT [130]. Consistent with these findings, Liang et al. illustrated that circ_0058063 promotes BCa cell proliferation and invasion by upregulating FOXP4 expression [76]. G3BP2, a member of the Ras-GTPase-activating protein (RasGAP) SH3 domain-binding protein (G3BP) family, is significantly overexpressed in multiple types of human tumours and contributes to tumour invasion [131, 132]. Circ_FNDC3B has been found to inhibit BCa cell proliferation, migration and invasion by suppressing the G3BP2 and SRC/FAK signalling pathways [133]. In contrast, androgen receptor (AR) has been found to mediate BCa development and progression [134, 135]. Chen et al. confirmed that AR-mediated circ_FNTA activity can promote BCa cell invasion via miR-370-3p/FNTA/KRAS signals [136]. Notably, the critical roles of cancer stem cells (CSCs) or cancer-initiating cells in tumorigenesis have attracted increasing scientific attention [137, 138]. Circ_103809 has been identified to be highly expressed in bladder CSCs and to promote the self-renewal, migration and invasion of BCa by sponging miR-511 [139]. Insulin-like growth factor-binding protein 2 (IGFBP2) has been found to be related to cell migration and invasion [140]. Yang et al. discovered that circ_VANGL1 accelerates BCa cell invasion, migration and proliferation by increasing IGFBP2 expression [141]. In another study, circ_VANGL1 was found to accelerate BCa cell invasion, migration and proliferation by increasing VANGL1 expression [77]. Liu et al. proposed that circ_DOCK1 increases the proliferation and migration potential of BCa cells via the circDOCK1/hsa-miR-132-3p/Sox5 signalling pathway [142]. Lin et al. demonstrated that circ_LPAR1 reduces invasion and metastasis via miR-762 [143]. Finally, Liu et al. verified that circ_UBXN7 suppresses cell growth and invasion by upregulating B4GALT3 [144].

Increasing evidence has revealed that circRNAs can act as metastasis activators or suppressors in BCa. The Wnt/β-catenin signalling pathway is highly involved in tumour metastasis [145, 146]. Han et al. illustrated that hsa_circ_0017247 enhances BCa cell metastasis by activating the Wnt/β-catenin signalling pathway [147]. In addition, Li et al. proposed that hsa_circ_0137606 can suppress BCa cell proliferation and metastasis via the hsa_circ_0137606/miR-1231/PHLPP2 axis [66]. Circ_ACVR2A has been found to significantly suppress the proliferation and metastasis of BCa by targeting the miR-626/EYA4 axis [148]. According to Wu et al. [149], circ_0023642 suppresses BCa cell invasion and metastasis by modulating the circ_0023642/miR-490-5p/EGFR signalling pathway. In addition, circ_UVRAG suppresses BCa cell proliferation and metastasis by targeting the miR-223/FGFR2 axis [150] (Fig. 4b). Li et al. found that circ_MTO1 inhibits BCa cell EMT and metastasis by sponging miR-221 [151]. FAK, a well-known tyrosine kinase, is closely related to metastasis in cancer [152]. Finally, circ_PICALM, which is generated from exons 9–12 of PICALM, has been identified to inhibit metastasis of BCa by modulating FAK activation and EMT [153].

#### Angiogenesis

Angiogenesis, the process by which rapidly growing malignant tissues are continuously supplied with nutrients and oxygen and cleared of metabolic wastes, is essential for tumour growth and progression. Without angiogenesis, tumours are unlikely to grow beyond a size of 100–200 μm [154]. Vascular endothelial growth factor (VEGF), a marker gene of angiogenesis, plays a key role in inducing angiogenesis during tumour growth and metastasis [155]. VEGFA, the expression of which is strongly induced by hypoxia, is one of the most potent inducers of angiogenesis [156]. VEGFR2, the primary VEGFA receptor, is the key molecule for VEGF signalling in tumour angiogenesis [157]. Circ_0001429 has been reported to induce angiogenesis to promote BCa cell growth and metastasis by increasing VEGFA expression [73]. As illustrated by Zhong et al. [69], circRNA_MYLK promotes angiogenesis by increasing the levels of VEGFA and the activity of VEGFR2. Circ_HIPK3 has also been demonstrated to inhibit angiogenesis of BCa cells by sponging miR-558 to reduce heparanase (HPSE) expression [64] (Fig. 4c). In addition, circ_403658, which is induced by HIF-1α, increases the expression of VEGFR and EGFR [158]. In addition to the VEGF family, the fibroblast growth factor (FGF) family is the other best-studied family of angiogenic growth factors. These factors could promote the proliferation, differentiation and migration of endothelial cells during angiogenesis by interacting with their corresponding receptors [159, 160]. According to Mao et al., the hsa_circ_0068871/miR-181a-5p/FGFR3 axis may play a vital role in the progression of BCa [161].

#### Cisplatin chemoresistance

Although BCa is relatively sensitive to chemotherapy, decreasing cisplatin chemoresistance is a crucial therapeutic strategy for MIBC [162, 163]. RAB27A, a member of the Rab family, plays pivotal roles in multiple processes of tumourigenesis via protein transport and small GTPase-mediated signal transduction [164]. Moreover, upregulation of RAB27A expression promotes proliferation and chemoresistance in BCa [165]. Consistent with these findings, Bi et al. found that circ-BPTF promotes BCa progression by increasing RAB27A expression [166]. APAF-1, a major apoptosis-regulating factor, has also been found to modulate cisplatin sensitivity [167–169]. Notably, Yuan et al. revealed that circ_Cdr1as may increase the cisplatin-induced chemosensitivity of BCa cells through the circ_Cdr1as/miR-1270/APAF1 axis [116]. Hypoxia also enhances resistance to therapy, thus playing critical roles in cancer biology [170]. Furthermore, cancer stem-like cells have been reported to contribute to cisplatin resistance in BCa [171]. Su et al. identified a specific hypoxia-elevated circRNA, circ_ELP3, that promotes cisplatin resistance in BCa by targeting cancer stem-like cells [172]. AR has also been found to mediate cisplatin sensitivity and thereby suppress BCa cell growth [173]. Indeed, AR-mediated circ_FNTA activity decreases cisplatin sensitivity via miR-370-3p/FNTA/KRAS signals [136].

#### Molecular mechanisms of circRNAs in BCa

CircRNAs perform regulatory roles mainly by acting as miRNA sponges [22], interacting with RBPs [23], and being translated into peptides [24]. Most circRNAs can regulate BCa-related signalling pathways via ceRNA-related regulatory mechanisms. The ceRNA hypothesis specifies that circRNAs can act as molecular sponges that compete with mRNAs for binding to miRNAs, thus inhibiting the activities of the corresponding miRNAs. miRNAs exert their functions through three mechanisms: (1) suppression of translation by binding to the 3’UTRs of target genes, (2) activation of translation by binding to the promoters of target genes, and (3) activation of translation by binding to the 5’UTRs of target genes. For example, circ_ITCH acts as a molecular sponge for miR-224 in BCa; as miR-224 normally inhibits PTEN expression by targeting its 3’UTR, circ_ITCH-mediated sponging ultimately leads to upregulation of PTEN expression in BCa [89]. In addition, circ_HIPK3 sponges miR-558, which normally directly binds to the promoter of the HPSE gene and increases its mRNA expression; thus, circ_HIPK3-mediated sponging in BCa ultimately negatively regulates HPSE expression [64]. Finally, Zheng et al. revealed that circ_NR3C1 directly sponges miR-27a-3p; as miR-27a-3p typically interacts with the cyclin D1 mRNA 5’UTR to facilitate nearby initiating ribosome binding, circ_NR3C1-mediated sponging downregulates cyclin D1 expression [109] (Fig. 4d). Similarly, circ_FNDC3B has been reported to suppress G3BP2 expression by sponging miR-1178-3p, which binds to the 5’UTR of G3BP2 [133]. It should be noted that the miRNA sponge function of circRNAs also depends on the abundance of miRNAs/circRNAs [174] and the number of binding sites for miRNAs contained in each cell [175]. CircRNAs containing many competing binding sites are more likely to have miRNA sponge functions [176, 177]. The most well-characterized circRNA is ciRS-7, which contains more than 70 miR-7 binding sites. It serves as a miR-7 sponge, leading to decreased miR-7 activity and accelerated expression of miR-7-targeted transcripts [178]. Many other circRNAs containing fewer miRNA binding sites can also serve as miRNA sponges. However, their miRNA sponging ability may be limited. Thus, it seems unlikely that all circRNAs can function as miRNA sponges.

### Clinical significance of circRNAs in BCa

**Fig. 5:**
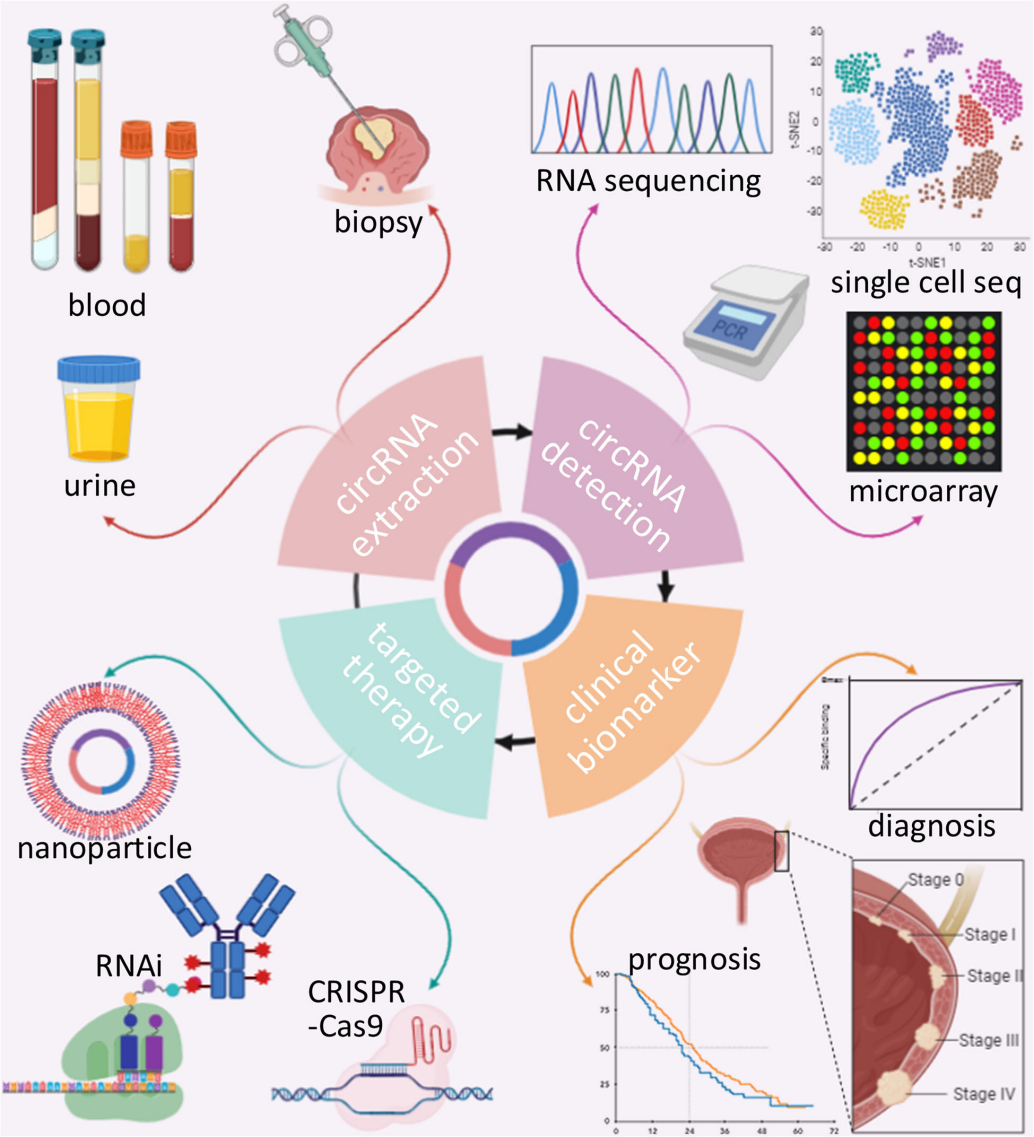
Clinical applications of circRNAs in BCa

The high incidence and mortality of BCa reflect the need for strategies to improve its early diagnosis, prognosis, and effective treatment. CircRNAs show considerable potential for use as diagnostic and prognostic biomarkers in BCa. First, circRNAs, as unique endogenous noncoding RNAs, are highly conserved and broadly expressed in various tissues, including human BCa and normal bladder tissues [37, 64, 179]. Second, circRNAs are characterized by high stability due to their covalently closed loop structures and by resistance to RNA exonucleases or RNase R [180]. Third, the expression profiles of circRNAs are cell type-specific, tissue-specific, or developmental stage-specific [9, 50]. Finally, apart from solid tissues, BCa-related circRNAs can be detected in blood and urine [81, 97]. RNA-seq [32], circRNA microarrays [50], PCR [51], and Northern blot analysis [57] are widely used methods for circRNA detection and identification. CircRNAs with potential diagnostic, prognostic and predictive value in BCa are summarized in Table 3. As mentioned above, circRNAs play crucial regulatory roles in BCa and are involved in various signalling pathways in BCa, including pathways related to cell proliferation, tumour growth suppression, cell cycle arrest, apoptosis, invasion, migration, metastasis, angiogenesis, and cisplatin chemoresistance. Thus, overexpression or knockdown of related circRNAs might be an effective intervention strategy for BCa progression. RNA interference (RNAi) [181–183], CRISPR/Cas9 editing [55], plasmid transfection [184], and lentiviral vector infection [185] are methods that can be used to decrease or increase circRNA levels. Additionally, nanoparticles can be loaded with exogenous circRNAs and used to carry them for targeted therapy [186] (Fig. 5).

**Table 3:** Utility of circRNAs for the clinical management of BCa

circRNA name	circBase ID	Cilinical Sample	Utility			Ref./PMID
			Diagnostic	Prognostic	Predictive	
circRNA-MYLK	hsa_circ_0002768	tissue		√		28687357
Circ_0058063	hsa_circ_0058063	tissue		√		32181485
hsa_circ_0076704	hsa_circ_0076704	tissue		√		31169020
hsa_circ_0000144	hsa_circ_0000144	tissue		√		30098434
circUVRAG	hsa_circ_0023642	tissue		√		30387298
circ_0071662	hsa_circ_0071662	tissue		√		31757227
circ-ITCH	hsa_circ_0001141	tissue		√		29386015
circLPAR1	hsa_circ_0087960	tissue		√		30867795
circPTPRA	hsa_circ_0006117	tissue		√		31821171
circUBXN7	hsa_circ_0001380	tissue		√		30312173
ciRs-6	hsa_circ_0006260	tissue		√		31819015
circ_FAM114A2	hsa_circ_0001546	tissue		√		31969560
circ_SLC8A1	hsa_circ_0000994	tissue		√		31228937
circ_0068871	hsa_circ_0068871	tissue		√		30999937
CEP128	hsa_circ_0102722	tissue			√	30134837
circPTK2	hsa_circ_0003221	tissue and blood			√	29125888
circCDYL	hsa_circ_0008285	tissue		√	√	29263845, 30968727
circHIPK3	hsa_circ_0000284	tissue		√	√	29263845, 28794202
circFUT8	hsa_circ_0003028	tissue		√	√	32072011
circRNA_403658	hsa_circ_0004383	tissue		√	√	31814891
circRNA_000285	hsa_circ_0000285	tissue and serum		√	√	30509102
circPICALM	hsa_circ_0023919	tissue		√	√	31648990
circ0001361	hsa_circ_0001361	tissue		√	√	31705065
circRIP2	hsa_circ_0005777	tissue		√	√	32019579
cTFRC	has-circ-0001445	tissue		√	√	30782157
circ-VANGL1	hsa_circ_0002623	tissue		√	√	30146736
circ5912	hsa_circ_0005912	tissue		√	√	31808751
circFNDC3B	hsa_circ_0006156	tissue		√	√	30458784
circ-ZKSCAN1	hsa_circ_0001727	tissue		√	√	31481066
circMTO1	hsa_circ_0007874	tissue		√	√	30551873
circCASC15	hsa_circ_0075828	tissue		√	√	31072448
Circ-BPTF	hsa_circ_0000799	tissue		√	√	30103209
circPRMT5	hsa_circ_0031250	tissue, serum and urine		√	√	30305293
hsa circ 0018069	hsa circ_0018069	tissue	√		√	30984788
circZFR	hsa_circ_0072088	tissue	√	√	√	31746333
circASXL1	hsa_circ_0001136	tissue	√	√	√	31966702
circ_0137439	hsa_circ_0137439	urine	√	√	√	31777254

#### Relationships between circRNA levels and clinicopathologic characteristics in BCa

CircRNAs have been reported to be significantly associated with many clinicopathologic characteristics in BCa, including tumour size, grade, differentiation, and stage; lymph node metastasis (LNM); tumour numbers; distant metastasis (DM); invasion; and recurrence. Li et al. observed that circ_0018069 is significantly downregulated in BCa tissues and in T24 and Biu-87 cells and that circ_0018069 levels are correlated with tumour grade, tumour stage, and muscular invasion depth in the context of BCa [80]. Circ_0137439 has also been reported to be significantly upregulated in urine samples from individuals with BCa. Moreover, hsa_circ_0137439 levels are correlated with tumour stage and grade, LNM, and history of MIBC [81]. Furthermore, circASXL1 is evidently upregulated in tissues obtained from BCa patients, and its levels are significantly associated with tumour grade, tumour stage, lymph node invasion, and DM [187]. Similarly, circ_ZFR is significantly upregulated in BCa tissues, and its levels are positively correlated with tumour stage, tumour grade, and LNM [82]. Chi et al. demonstrated that hsa_circ_0000285 levels are significantly reduced in BCa tissues and serum compared to adjacent tissues and serum from healthy controls and that this downregulation is associated with cisplatin resistance, tumour size, differentiation, LNM, DM, and TNM stage [83]. Circ_0001361 has been shown to be overexpressed in BCa tissues and cell lines, and its levels in BCa tissues are correlated with pathologic grade and muscle invasion [124]. Similarly, hsa_circ_0068871 is overexpressed in BCa tissues and cell lines, and its levels in BCa tissues are correlated with T stage and N stage [161]. In contrast, circ_0071662 is downregulated in BCa tissues and cell lines, and its expression levels are significantly associated with LNM and DM [94]. Zhuang et al. revealed that high hsa_circ_0075828 expression in BCa tissues and cells is associated with tumour stage [86]. According to Su and colleagues, circ_5912 is significantly downregulated in BCa tissues compared with normal control tissues, and its levels are correlated with BCa grade, stage, and metastasis [126]. The expression levels of circ_BPTF have been found to be increased in BCa tissues and cell lines compared with noncancerous tissues and cell lines, and high levels of circ_BPTF are positively associated with tumour grade [166]. Sun et al. suggested that circ_CDYL is expressed at low levels in BCa tissues and cell lines and that its expression levels are negatively correlated with BCa pathological stage [107]. In contrast, circ_CEP128 is significantly upregulated in BCa tissues, and its levels correlate positively with tumour size, TNM stage and LNM [74]. Circ_FAM114A2 has been identified to be downregulated in both BUC tissue specimens and cell lines, and high circ_FAM114A2 expression levels are negatively associated with pathological TNM stage and grade [99]. Similarly, circ_FNDC3B is downregulated in BC tissues, and its levels correlate with pathological T stage, grade, and LNM [133]. In addition, other circRNAs, such as circ_FUT8 [127], circ_HIPK3 [64, 68], circ_ITCH [89], circ_MTO1 [151], circ_PICALM [153], circ_PRMT5 [62], circ_PTK2 [97], circ_PTPRA [101], circ_RIP2 [128], hsa_circ_0058063 [76], circ_403658 [158], circ_SLC8A1 [88], circ_TFRC [129], circ_UBXN7 [144], circ_VANGL1 [77], circ_ZKSCAN1 [111], and ciRs_6 [103], are also detectable in BCa tissue or blood and are associated with various clinicopathologic characteristics in BCa (Table 4). Most studies have reported that there is no relationship between circRNA levels and gender in BCa. It should be noted that epidemiological studies show obvious gender differences in the incidence and prognosis of BCa [134]. The aetiology of this gender difference has been linked to sex hormones and their receptors, including estrogen receptor (ER) and AR [188, 189]. Circ_0023642 and circ_FNTA are estrogen receptor- and androgen receptor-mediated circRNAs, respectively. Wu et al. found that estrogen receptor alpha (ERα) decreased circ_0023642 levels and subsequently increased miR-490-5p expression, resulting in decreased EGFR expression to suppress BCa cell invasion [149]. Similarly, Chen et al. reported that the AR-regulated circular RNA circFNTA competes with the microRNA miR-370-3p to increase the expression of its host gene FNTA, which then activates KRAS signalling to promote BCa cell invasion and resistance to cisplatin [136].

**Table 4:** Relationship between circRNAs level and clinicopathologic characteristics in BCa

Study	circRNA Name	CircBase ID	Host gene	Position	Cilinical Sample	Detection method	Cut-off value	Reference gene	Dysregulation	Number of patients	Gender	Age	Tumor size	Grade	DF	Stage	LMN	Number of tumors	DM	Invasion	Recurrence	Potential function	AUC	Sensitivity and specifcity	OS	DFS/RFS	PFS	Ref./PMID
Li et al.	circ_0018069	hsa circ_0018069	KIAA1462	chr10:30315031–30318795	tissues	qPCR	0.0007	β-actin	downregulated	41	No	No	No	Yes	–	Yes	No	–	–	Yes	–	Diagnosis	0.709	97.6, 46.3%	–	–	–	30984788
Song et al.	circ_0137439	hsa_circ_0137439	MTDH	chr8:98673299–98708521	urine	qPCR	1.36	GAPDH	upregulated	116	No	No	–	Yes	–	Yes	Yes	–	–	Yes	–	Diagnosis	0.89	87.93, 80.06%	Yes	Yes	–	31777254
Tang et al.	circ_ASXL1	hsa_circ_0001136	ASXL1	chr20:30954186–30956926	tissues	qPCR	–	U6	upregulated	61	No	No	No	Yes	–	Yes	Yes	No	Yes	No	–	Diagnosis	0.77	68.6, 76.9%	Yes	–	–	31966702
Zhang et al.	circ_ZFR	hsa_circ_0072088	ZFR	chr5:32379220–32388780	tissues	qPCR	MEL	GAPDH	upregulated	104	No	No	No	Yes	–	Yes	Yes	–	–	No	Yes	Diagnosis	0.8216	–	Yes	–	Yes	31746333
Huang et al.	circ_0000144	hsa_circ_0000144	SLAMF6	chr1:160472466–160472794	tissues	qPCR	–	U6	upregulated	69	–	–	–	–	–	–	–	–	–	–	–	–	–	–	Yes	–	–	30098434
Chi et al.	circ_000285	hsa_circ_0000285	HIPK3	chr11:33362513–33363232	tissues, serum	qPCR	MV	β-actin	downregulated	197	No	No	Yes	–	Yes	Yes	Yes	–	Yes	–	–	–	–	–	Yes	–	–	30509102
Liu et al.	circ_0001361	hsa_circ_0001361	FNDC3B	chr3:171830241–171851336	tissues	qPCR	–	GAPDH	upregulated	69	No	No	No	Yes	–	Yes	No	–	–	Yes	–	–	–	–	Yes	–	–	31705065
Mao et al.	circ_0068871	hsa_circ_0068871	FGFR3	chr4:1801473–1804791	tissues	qPCR	–	GAPDH	upregulated	32	No	No	No	–	–	Yes	–	–	No	–	–	–	–	–	–	–	–	30999937
Abulizi et al.	circ_0071662	hsa_circ_0071662	TPPP	chr5:659976–693510	tissues	qPCR	–	–	downregulated	158	No	No	Yes	–	–	Yes	–	–	–	–	–	–	–	–	Yes	–	–	31757227
Zhuang et al.	circ_CASC15	hsa_circ_0075828	CASC15	chr6:22020567–22020771	tissues	qPCR	–	GAPDH	upregulated	67	No	No	No	–	–	Yes	No	–	–	–	–	–	–	–	Yes	Yes	–	31072448
Su et al.	circ_5912	hsa_circ_0005912	FIP1L1	chr4:54265896–54294350	tissues	qPCR	–	GAPDH	downregulated	58	No	No	–	Yes	–	Yes	Yes	No	–	–		–	–	–	Yes	–	–	31808751
Bi et al.	circ_BPTF	hsa_circ_0000799	BPTF	chr17:65941524–65972074	tissues	qPCR	MEL	–	upregulated	72	No	No	No	Yes	–	No	No		–	Yes	Yes	–	–	–	Yes	–	–	30103209
Sun et al.	circ_CDYL	hsa_circ_0008285	CDYL	chr6:4891946–4892613	tissues	qPCR	–	GAPDH	downregulated	30	No	No	–	No	–	Yes	No		–	No		–	–	–	–	–	–	30968727
Okholm et al.	circ_CDYL	hsa_circ_0008285	CDYL	chr6:4891946–4892613	tissues	qPCR	MEL	–	downregulated	457	–	–	–	–	–	–	–	–	–	–	–	–	–	–	–	–	Yes	29263845
Wu et al.	circ_CEP128	hsa_circ_0102722	CEP128	chr14:81209418–81304622	tissues	qPCR	–	GAPDH	upregulated	10	No	No	Yes	–	–	Yes	Yes	–	–	–		–	–	–	–	–	–	30134837
Liu et al.	circ_FAM114A2	hsa_circ_0001546	FAM114A2	chr5:153413351–153414527	tissues	qPCR	NAT	GAPDH	downregulated	31	No	No	No	Yes	–	Yes	No	–	–	–		–	–	–	–	–	–	31969560
Liu et al.	circ_FNDC3B	hsa_circ_0006156	FNDC3B	chr3:171965322–171969331	tissues	qPCR	–	GAPDH	downregulated	82	No	No	No	Yes	–	Yes	Yes	–	–	No	No	–	–	–	Yes	–	–	30458784
He et al.	circ_FUT8	hsa_circ_0003028	FUT8	chr14:66028054–66028484	tissues	qPCR	–	GAPDH	downregulated	145	No	No	No	Yes	–	No	Yes	No	–	–	–	–	–	–	Yes	–	–	32072011
Li et al.	circ_HIPK3	hsa_circ_0000284	HIPK3	chr11:33307958–33309057	tissues	qPCR	–	GAPDH	downregulated	44	No	No	–	Yes	–	Yes	Yes	–	–	Yes	–	–	–	–	–	–	–	28794202
Okholm et al.	circ_HIPK3	hsa_circ_0000284	HIPK3	chr11:33307958–33309057	tissues	qPCR	MEL	–	downregulated	457	–	–	–	–	–	–	–	–	–	–	–	–	–	–	–	–	Yes	29263845
Yang et al.	circ_ITCH	hsa_circ_0001141	ITCH	chr20:33001547–33037285	tissues	qPCR	–	β-actin	downregulated	70	No	No	No	Yes	–	No	–	–	–	–	–	–	–	–	Yes	–	–	29386015
Lin et al.	circ_LPAR1	hsa_circ_0087960	LPAR1	chr9:113734352–113735838	tissues	qPCR	–	β-actin	upregulated	125	No	No	No	No	–	No	–	No	–	–	No	–	–	–	–	–	–	30867795
Li et al.	circ_MTO1	hsa_circ_0007874	MTO1	chr6:74175931–74176329	tissues	qPCR	–	β-actin	downregulated	117	–	–	–	–	–	–	Yes	–	–	–	–	–	–	–	Yes	Yes	–	30551873
Yan et al.	circ_PICALM	hsa_circ_0023919	PICALM	chr11:85707868–85714494	tissues	qPCR	–	GAPDH	downregulated	168	No	No	No	Yes	–	Yes	Yes	–	–	–	–	–	–	–	Yes	–	–	31648990
Chen et al.	circ_PRMT5	hsa_circ_0031250	PRMT5	chr14:23395341–23396023	tissues	qPCR	–	GAPDH	upregulated	119	No	No	–	No	–	Yes	–	–	–	–	–	–	–	–	–	Yes	–	30305293
Xu et al.	circ_PTK2	hsa_circ_0003221	PTK2	chr8:141856358–141900868	tissue, blood	qPCR	–	GAPDH	upregulated	40	–	–	–	–	Yes	Yes	Yes	–	–	–	–	–	–	–	–	–	–	29125888
He et al.	circ_PTPRA	hsa_circ_0006117	PTPRA	chr20:2944917–2945848	tissues	qPCR	–	GAPDH	downregulated	104	No	No	Yes	No	–	Yes	No	No	–	–	–	–	–	–	Yes	–	–	31821171
Su et al.	circ_RIP2	hsa_circ_0005777	RGNEF	chr5:73136304–73136585	tissues	qPCR	–	GAPDH	downregulated	58	No	No	–	Yes	–	Yes	Yes	No	–	–	–	–	–	–	Yes	–	–	32019579
Liang et al.	circ_0058063	hsa_circ_0058063	ATIC	chr2:216177220–216213972	tissues	qPCR	–	GAPDH	upregulated	94	No	No	–	Yes	–	Yes	–	No	–	–	–	–	–	–	Yes	–	–	32181485
Wei et al.	circ_403658	hsa_circ_0004383	ZNF292	chr6:87920168–87928449	tissues	qPCR	–	GAPDH	upregulated	123	No	No	Yes	–	–	Yes	No	–	Yes	–	–	–	–	–	Yes	–	–	31814891
Lu et al.	circ_SLC8A1	hsa_circ_0000994	SLC8A1	chr2:40655612–40657444	tissues	qPCR	–	GAPDH	downregulated	70	No	No	–	Yes	–	Yes	No	–	–	–	–	–	–	–	–	–	–	31228937
Su et al.	circ_TFRC	has_circ_0001445	TFRC	chr3:195785154–195,787,118	tissues	qPCR	–	GAPDH	upregulated	220	No	No	–	Yes	–	Yes	Yes	No	–	–	–	–	–	–	Yes	–	–	30782157
Liu et al.	circ_UBXN7	hsa_circ_0001380	UBXN7	chr3:196118683–196129890	tissues	qPCR	–	GAPDH	downregulated	84	No	No	No	Yes	–	Yes	No	–	–	No	–	–	–	–	Yes	–	–	30312173
Zeng et al.	circ_VANGL1	hsa_circ_0002623	VANGL1	chr1:116202261–116206889	tissues	qPCR	–	GAPDH	upregulated	43	No	No	–	Yes	–	Yes	Yes	–	–	–	–	–	–	–	Yes	–	–	30146736
Bi et al.	circ_ZKSCAN1	hsa_circ_0001727	ZKSCAN1	chr7:99621041–99621930	tissues	qPCR	–	GAPDH	downregulated	137	No	No	No	Yes	–	Yes	Yes	–	–	–	Yes	–	–	–	Yes	Yes	–	31481066
Su et al.	ciRs_6	hsa_circ_0006260	SLC41A2	chr12:105321750–105322472	tissues	qPCR	–	GAPDH	downregulated	58	No	No	–	Yes	–	Yes	No	No	–	–	–	–	–	–	Yes	–	–	31819015
Liu et al.	circ_0076704	hsa_circ_0076704	CD2AP	chr6:47471015–47522502	tissues	qPCR	–	β-actin	upregulated	70	–	–	–	–	–	–	–	–	–	–	–	–	–	–	Yes	–	–	31169020
Yang et al.	circ_UVRAG	hsa_circ_0023642	UVRAG	chr11:75727858–75728024	tissues	qPCR	–	–	upregulated	402	–	–	–	–	–	–	–	–	–	–	–	–	–	–	NO	–	–	30387298
Zhong et al.	circ_MYLK	hsa_circ_0002768	MYLK	chr3:123471177–123512691	tissues	qPCR	–	–	upregulated	32	–	–	–	–	–	–	–	–	–	–	–	–	–	–	Yes		–	28687357

*circRNAs* circular RNAs, *BCa* bladder cancer, *MV* mean value, *MEL* median expression level, *LNM* lymph nodes metastasis, *NAT* adjacent noncancerous tissues, *DF* differentiation, *LNM* lymph node metastasis, *DM* distant metastasis, *AUC* area under the curve, *OS* over survival, *DFS* disease-free survival, *RFS* recurrence-free survival, *PFS* progression-free survival

#### CircRNAs as diagnostic biomarkers for BCa

The clinical value of circRNAs as diagnostic biomarkers has been explored in many studies. The area under the receiver operating characteristic (ROC) curve (AUC) of circ_0018069 for BCa diagnosis is 0.709, and the sensitivity and specificity are 97.6 and 46.3%, respectively [80]. The AUC of a ROC curve generated for urinary cell-free hsa_circ_0137439 levels is 0.890, with a sensitivity and specificity of 87.93 and 80.06%, respectively [81]. The AUC for circASXL1 in tumour invasion (T2-T4 tumour) diagnosis is 0.770, with a sensitivity and specificity of 68.6 and 76.9%, respectively [187]. The AUC for circ_ZFR in BCa diagnosis is 0.8216 [82] (Table 4).

#### CircRNAs as prognostic biomarkers for BCa

CircRNA levels can also be used to predict patient survival parameters, such as overall survival (OS), disease-free survival (DFS), and progression-free survival (PFS). To further analyse the prognostic value of circRNAs in BCa, we collected information from studies reporting survival information and evaluated the associations between circRNA expression levels and OS, DFS, and PFS. Fourteen upregulated circRNAs were reported to predict poor OS [69–71, 76, 77, 81, 82, 86, 124, 129, 150, 158, 166, 187], while thirteen downregulated circRNAs were reported to predict poor OS [83, 89, 94, 101, 103, 111, 126–128, 133, 144, 151, 153]. Kaplan-Meier survival analysis indicated that higher expression of circ_0137439, circ_CASC15, and circPRMT5 was associated with poorer DFS [62, 81, 86]. Two studies revealed that higher expression of circ_ZKSCAN1 and circ_MTO1 was associated with longer DFS [111, 151]. A study by Zhang et al. revealed a significantly elevated risk of progression for patients with high circ_ZFR expression levels [82]. In addition, patients with high circ_CDYL and circ_HIPK3 expression were reported to have a reduced risk of progression [68].

## Conclusion

Over the past 10 years, the importance of elucidating circRNA biology to our understanding of tumorigenesis has become evident. As outlined in this review, considerable evidence indicates that circRNAs play key roles in BCa. To date, fifty-five circRNAs among hundreds of aberrantly expressed circRNAs have been identified to be specifically associated with BCa. Notably, BCa-related circRNAs have been discovered to regulate cancer-related biological behaviours via ceRNA regulatory mechanisms. Existing reports feature methodologies and study designs that others can use for further investigation of circRNAs of interest. CircRNAs have been reported to be significantly associated with many clinicopathologic characteristics of BCa and with BCa patient survival parameters, and the abundance, conservation, stability, specificity and detectability of circRNAs render them potential diagnostic and prognostic biomarkers for BCa. Additionally, circRNAs play crucial regulatory roles upstream of various signalling pathways related to BCa carcinogenesis and progression, reflecting their potential as therapeutic targets for BCa.

Some limitations of previous research on circRNAs in BCa should be noted. First, the biogenesis of circRNAs and the regulatory mechanisms involved in circularization remain vague. More research is needed to help us understand the circRNA circularization processes in depth. Second, no unified standards are available to determine thresholds for circRNA detection. Third, previous studies on circRNAs in BCa lacked circRNAs with BCa specificity. More circRNAs with relative bladder cancer specificity may be further characterized in future studies. Fourth, almost all reported circRNAs in BCa exert functions via miRNA sponge mechanisms. The other three classical mechanisms, including sponging of RBPs, regulation of transcription and translation into peptides or proteins, have rarely been studied in BCa. Fifth, all circRNAs reported in BCa are currently in the basic research stage. Further investigation of circRNAs as diagnostic biomarkers, prognostic biomarkers, or targeted therapy for BCa in well-designed multicentre cohort studies is necessary.

## Supplementary Information

**Additional file 1: Table S1.** Database for circRNA research

## Data Availability

Not applicable.

## Abbreviations

circRNAs: Circular RNAs
BCa: Bladder cancer
OS: Overall survival
DFS: Disease-free survival
PFS: Progression-free survival
ecRNAs: Exonic circRNAs
elciRNAs: Exon-intron circRNAs
ciRNAs: Intronic circRNAs
miRNA: microRNA
RBPs: RNA-binding proteins
BUC: Bladder urothelial carcinoma
MIBC: Muscle-invasive bladder cancer
NMIBC: Non-muscle-invasive bladder cancer
QKI: Quaking
MBL: Muscleblind
ADAR1: Adenosine deaminase acting on RNA
Cul2: Cullin2
HuR: Human antigen R/ELAV-like protein 1
IRESs: Internal ribosome entry sites
BSJs: Back-splice junctions
FISH: Fluorescence in situ hybridization
RNA-seq: RNA sequencing
RT-PCR: Reverse transcription-polymerase chain reaction
GEO: Gene Expression Omnibus
FDR: False discovery rate
PTEN: Phosphatase and tensin homologue
KLF9: Krüppel-like factor 9
APAF-1: Apoptosis protease-activating factor-1
FOXO3: Forkhead box transcription factor class O3
EMT: Epithelial-mesenchymal transition
G3BP: Ras-GTPase-activating protein SH3 domain-binding protein
AR: Androgen receptor
CSCs: Cancer stem cells
IGFBP2: Insulin-like growth factor-binding protein 2
VEGF: Vascular endothelial growth factor
HPSE: Heparanase
FGF: Fibroblast growth factor
RNAi: RNA interference
LNM: Lymph node metastasis
DM: Distant metastasis
ER: Estrogen receptor
ERα: Estrogen receptor alpha
ROC: Receiver operating characteristic
AUC: Area under the receiver operating characteristic curve
