# Supplementary material for: Expression profiles, biological functions and clinical significance of circRNAs in bladder cancer
Source: Mol Cancer. 2021 Jan 4;20:4. doi: 10.1186/s12943-020-01300-8 (PMC7780637; doi:10.1186/s12943-020-01300-8)
Supplement: Supplementary file 1 — Additional file 1: Table S1. Database for circRNA research [file 12943_2020_1300_MOESM1_ESM.doc]

Table S1. Database for circRNA research.

| Database | URL | Function | Ref./PMID |
| --- | --- | --- | --- |
|
| Circbase | http://www.circbase.org/ | Database contains circRNA information from different species. | 25234927 |
| CircRNADisease | http://cgga.org.cn:9091/circRNADisease/ | Database provides disease-associated circRNAs annotation. | 29700306 |
| Circ2Traits | http://gyanxet-beta.com/circdb/ | Database provides circRNAs that may be associated with disease in human. | 24339831 |
| CSCD | http://gb.whu.edu.cn/CSCD | Database contains cancer-specific circRNA annotation, predicting the microRNA response element sites and RNA binding protein sites for each circRNA, and predicting potential open reading frames for highlight translatable circRNAs. | 29036403 |
| TSCD | http://gb.whu.edu.cn/TSCD/ | Database deposits the features of tissue-specific (TS) circRNAs in human and mouse. | 27543790 |
| ExoRBase | http://www.exoRBase.org | Database provides annotation, expression level and possible original tissues of human blood exosome-derived circRNAs. | 30053265 |
| CircBank | http://www.circbank.cn | Database implements a novel naming system of circRNAs based on the host genes of circRNA, and provides the miRNA binding site, conservation of circRNAs, m6A modification of circRNAs, mutation of circRNAs and protein-coding potential of circRNAs. | 31023147 |
| CircPro | http://bis.zju.edu.cn/CircPro/ | Database detects circRNAs with protein-coding potential from high-throughput sequencing data, and provides the information of genomic position, type and junction reads from RNA-Seq and Ribo-Seq, and so on. |  |
| Starbase v2.0 | http://starbase.sysu.edu.cn/ | Database predicts circRNA-miRNA interactions. | 24297251 |
| CircNet | http://circnet.mbc.nctu.edu.tw/ | Database provides novel circRNAs, integrated miRNA-target networks, expression profiles of circRNA isoforms, genomic annotations of circRNA isoforms, and sequences of circRNA isoforms. | 26450965 |
| deepBase v2.0 | http://rna.sysu.edu.cn/deepBase/ | Database annotates 14 867 human circRNAs. | 26590255 |
| CircRNADb | http://reprod.njmu.edu.cn/circrnadb | Database provides genomic information, exon splicing, genome sequence, internal ribosome entry site (IRES), open reading frame (ORF) and references for circRNAs. | 27725737 |
| CircInteractome | http://circinteractome.nia.nih.gov/ | Database maps RBP (RNA-binding protein)-and miRNA-binding sites on human circRNAs. | 26669964 |
| CIRCpedia v2 | http://www.picb.ac.cn/rnomics/circpedia | Database containes comprehensive circRNA annotation from over 180 RNA-seq datasets across six different species. | 30172046 |
| CirclncRNAnet | http://app.cgu.edu.tw/circlnc/ | Database provides a "one-stop" resource for in-depth analyses of ncRNA biology. | 29194536 |
| TRCirc | http://www.licpathway.net/TRCirc | Database provides transcriptional regulation information of circRNAs. | 30184150 |

URL, uniform resource locator.
